# Radiopharmaceuticals for Treatment of Adrenocortical Carcinoma

**DOI:** 10.3390/ph17010025

**Published:** 2023-12-23

**Authors:** Kerstin Michalski, Wiebke Schlötelburg, Philipp E. Hartrampf, Aleksander Kosmala, Andreas K. Buck, Stefanie Hahner, Andreas Schirbel

**Affiliations:** 1 Department of Nuclear Medicine, Würzburg University Hospital, University of Würzburg, Oberdürrbacher Straße 6, D-97080 Würzburg, Germanybuck_a@ukw.de (A.K.B.); schirbel_a@ukw.de (A.S.); 2 Division of Endocrinology and Diabetes, Department of Medicine I, Würzburg University Hospital, University of Würzburg, Oberdürrbacher Straße 6, D-97080 Würzburg, Germany; hahner_s@ukw.de

**Keywords:** adrenocortical carcinoma, theranostics, endoradiotherapy, IMAZA

## Abstract

Adrenocortical carcinoma (ACC) represents a rare tumor entity with limited treatment options and usually rapid tumor progression in case of metastatic disease. As further treatment options are needed and ACC metastases are sensitive to external beam radiation, novel theranostic approaches could complement established therapeutic concepts. Recent developments focus on targeting adrenal cortex-specific enzymes like the theranostic twin [^123/131^I]IMAZA that shows a good image quality and a promising therapeutic effect in selected patients. But other established molecular targets in nuclear medicine such as the C-X-C motif chemokine receptor 4 (CXCR4) could possibly enhance the therapeutic regimen as well in a subgroup of patients. The aims of this review are to give an overview of innovative radiopharmaceuticals for the treatment of ACC and to present the different molecular targets, as well as to show future perspectives for further developments since a radiopharmaceutical with a broad application range is still warranted.

## 1. Introduction

Adrenocortical carcinoma (ACC) is a rare tumor entity with an estimated incidence of about 0.5–2 new cases per million people per year [1,2]. ACC occurs at any age and shows a peak incidence between 40 and 60 years, whereby women are more often affected (55–60%) [3]. The tumor arises from the cortex of the adrenal gland and 50–60% of patients with ACC have clinical hormone excess. Treatment options are limited and complete resection is the only means of cure. Still, retrospective studies reported that 40–70% of ACCs eventually recur even after complete resection [4,5,6,7]. In general, the prognosis is heterogeneous and the median overall survival of all ACC patients is about 3–4 years. For tumors confined to the adrenal gland five-year survival rates are between 60–80%, for locally advanced disease 35–50%, and much lower in case of metastases with reported survival rates ranging from 0 to 28% [8,9,10,11,12,13,14].

Due to the rareness of the disease and the limited resources dedicated to the implementation of new therapeutic options, there is little progress in the medical therapy of ACC [15]. International guidelines recommend to use of adjuvant mitotane in most patients [3,16]. The results of a large phase 3 trial led to a combination treatment of mitotane, etoposide, doxorubicin, and cisplatin as a first-line therapy [11]. Unfortunately, the combination of these chemotherapeutics only led to an objective response rate of 23% with a progression-free survival of only 5.1 months despite severe toxicity. Hence, further therapeutic options for second- and third-line treatment are warranted. ACC used to be considered resistant to radiation [17,18]. However, recent data show a benefit in regards to local tumor control, the palliative treatment of symptomatic cerebral or osseous metastases and in case of vena cava obstruction as well as a reduction of local recurrence after primary resection [19,20,21,22,23,24,25]. In this sense, endoradiotherapy is a possible therapeutic option in patients with metastasized ACC after first-line treatment. The concept of endoradiotherapy is based upon theranostic radiopharmaceuticals that can be used for diagnostic and therapeutic purposes, depending on the labeled radionuclide. It is possible to use either the same molecule or a very similar compound. These molecules are radiolabeled with gamma and positron emitters for imaging purposes or beta minus emitters and (rarer) alpha emitters for endoradiotherapy. Some radionuclides, such as iodine-131 and lutetium-177 are beta and gamma emitters and can be used for both imaging and therapy, whereas the gamma emitter iodine-123 can be used only for diagnostics [26]. Other radionuclides for imaging are fluorine-18 or gallium-68 (both positron emitters) and Yttrium-90 (beta minus emitter) for therapy. The use of an image-based patient selection allows for a personalized medicine approach with a possible higher therapeutic efficacy. Furthermore, reduced side effects and high tumor doses can be administered because of the precise radiation deposition and the short tissue penetration of only a few millimeters of beta minus emitters [27].

The present review aims to give an overview of theranostic radiopharmaceuticals for the treatment of ACC, to present various molecular targets and to show future perspectives.

## 2. Molecular Imaging and Theranostic Approaches in ACC

For molecular imaging of ACC, positron emission tomography (PET)/computed tomography (CT) with [^18^F]fluorodeoxyglucose (FDG) can be used [28] but is not considered standard of care [3], in contrast to CT or magnetic resonance imaging. Nevertheless, FDG PET/CT is useful for prognostic evaluation as a higher uptake is associated with a shorter survival [29,30]. However, FDG does not provide a theranostic approach. For a detailed description of molecular imaging approaches in ACC, please refer to a recent review of adrenal imaging [31].

Peptide receptor radionuclide therapy targeting the somatostatin receptor (SSTR) using, i.e., [^177^Lu]Lu-DOTA-0-Tyr3-Octreotate (DOTATATE) is established in the treatment of well-differentiated neuroendocrine midgut tumors [32] and other neuroendocrine tumors [33]. A recent ex vivo study described a heterogeneous SSTR expression in some ACC tissue samples [34]. However, to date, only one study exists that reports the results of a case series of 19 patients with 2 patients receiving either [^90^Y]Y- or [^177^Lu]Lu-DOTATOC (DOTA(0)-Phe(1)-Tyr(3))octreotid), which resulted in disease control of 4 and 12 months, respectively [35].

In analogy, endoradiotherapy targeting the prostate-specific membrane antigen (PSMA) is not just a treatment option for metastasized castration-resistant prostate cancer using, i.e., [^177^Lu]Lu-vipivotide tetraxetan (PSMA-617) [36], but also for other tumor entities. In an ex vivo analysis, PSMA was significantly overexpressed in ACC tissue samples compared to normal adrenal glands and adrenocortical adenomas [37]. To our knowledge, there is no report providing data on PSMA radioligand therapy in ACC. Only one case report describes a patient with ACC having a PSMA expression in tumor sites equal to physiological liver background on [^68^Ga]Ga-PSMA-11 PET/CT, which was not considered sufficient for PSMA-directed radioligand therapy [38].

C-X-C motif chemokine receptor 4 (CXCR4) is a G-protein coupled receptor that can be found in many hematological malignancies as well as solid tumors and constitutes a possible theranostic target [39]. CXCR4 expression can be found in ACC samples as well [40]. A strong membranous expression of CXCR4 in ACC specimens was found in half of the cases (94 of 187 specimens) in an ex vivo study. Interestingly, immunohistochemical staining of CXCR4 was higher in samples derived from metastases than from primary tumors [41]. A high in vivo CXCR4 expression on CXCR4-directed PET/CT was found in 30 patients with ACC [42]. A possible theranostic application was found by Bluemel et al., who rated 17 (57%) of 30 patients as suitable and 4 patients (13%) as potentially suitable for CXCR4-directed treatment [43]. Of note, CXCR4-directed therapy using, i.e., [^177^Lu]Lu-/[^90^Y]Y-anditixafortide (PentixaTher) leads to bone marrow ablation and can only be applied in case of available hematopoietic stem cells which are usually harvested during previous chemotherapeutic protocols [44].

The enzymes CYP11B1 (11β-hydroxylase) and CYP11B2 (aldosterone synthase) are part of the cortisol and aldosterone synthesis in the adrenal gland and can be blocked by imidazole drugs such as etomidate or ketoconazole [45]. As these enzymes are highly specific for the adrenal gland, they are potential targets for molecular imaging [46]. Bergström et al. developed the PET imaging agent [^11^C]etomidate and its methyl ester [^11^C]metomidate ([^11^C]MTO) and showed their potential to specifically visualize the normal adrenal cortex in an animal study [47]. This approach was transferred to a clinical setting and the authors could demonstrate that [^11^C]MTO PET can distinguish between lesions of adrenocortical and nonadrenocortical origin in a cohort of 15 patients [48], and in another cohort of 173 patients [49]. The latter study included 13 patients with ACC which showed a relatively high tracer uptake.

**Figure 1:**
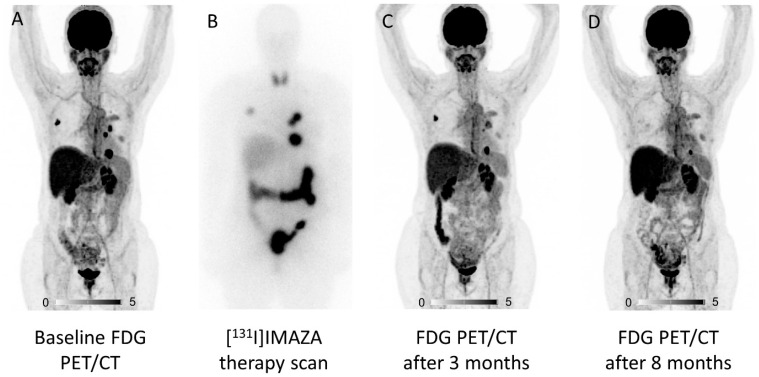
[^131^I]IMAZA therapy in a 53-year-old patient with metastatic adrenocortical cancer. FDG PET maximum intensity projection (MIP) is shown at baseline (**A**). Post-therapeutic whole-body scintigraphy 2 days after first therapy (**B**) shows concordant tracer accumulation to FDG PET/CT. Response assessment after 3 and 8 months (**C**,**D**) shows a significant decrease in metabolic activity and a reduction in the diameter of the target lesion of 26%. After a progression-free survival of 18 months, a second therapy with [^131^I]IMAZA was applied. The patient died after an overall survival of 56 months after the first [^131^I]IMAZA therapy.

**Figure 2:**
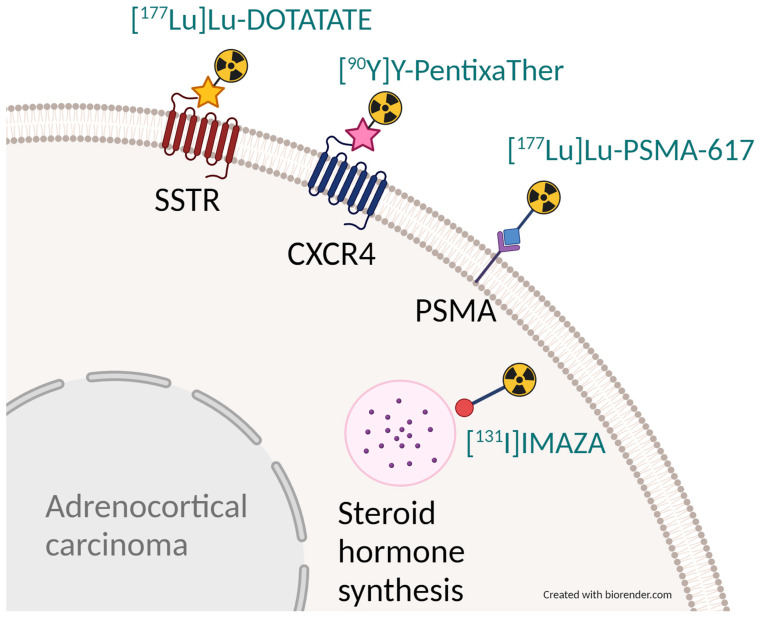
Schematic depiction of possible theranostic targets in adrenocortical carcinoma (black font) and the respective therapeutic radiopharmaceuticals (exemplary, blue font).

**Figure 3:**
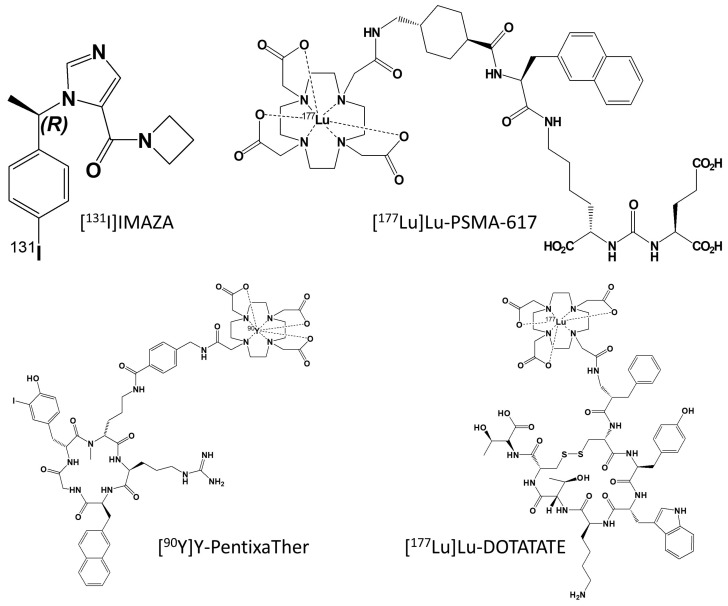
Chemical structure of possible theranostic radiopharmaceuticals for treatment of ACC. To date, [^131^I]IMAZA is the only compound that has been already used in patients.

In order to develop a possible theranostic radiopharmaceutical, the compound [^123^I]iodometomidate ([^123^I]IMTO) that inhibits CYP11B1/2 was developed. High imaging quality was shown in animal studies [50,51,52] and a high and specific tracer uptake of the radiopharmaceutical was found for adrenocortical tissue [51]. These promising results could be transferred into clinical application: [^123^I]IMTO planar whole-body scans and single photon emission computed tomography (SPECT)/CT images showed high sensitivity and specificity for the differentiation of adrenocortical tumors from lesions of non-adrenocortical origin in case of a lesion size of 2 cm or more [53]. The theranostic counterpart of [^123^I]IMTO is [^131^I]IMTO, which can be used in patients with advanced ACC. Disease control was achieved in 6 of 11 patients with ACC treated with [^131^I]IMTO with a median progression-free survival of 14 months (range 5–33 months) in responders. Of these, 5 patients showed a stable disease on follow-up CT scans, and a partial response was found in one patient [54]. As IMTO shows a rapid metabolic inactivation, the metabolically more stable derivative (R)-1-[1-(4-iodophenyl)ethyl]-1H-imidazole-5-carboxylic acid azetidinyl amide (IMAZA) was developed by replacing the methyl ester in IMTO by a carboxylic amide. IMAZA outperformed IMTO in regards to pharmacokinetic and imaging properties in mice and in a dual tracer approach in three patients [55]. Hahner et al. screened 69 patients with advanced ACC refractory to standard treatments using [^123^I]IMAZA SPECT/CT and identified 13 patients with intense uptake in all tumor lesions [56]. These patients were treated with a median of 25.7 GBq [^131^I]IMAZA (range 18.1–30.7 GBq). Response to therapy was assessed according to Response Evaluation Criteria in Solid Tumors (RECIST version 1.1) [57]. Two patients experienced a decrease in RECIST target lesions of up to 26%. A median progression-free survival of 14.3 months (range 8.3–21.9) was noted for five patients with stable disease. Median overall survival in all 13 patients was 14.1 months (4.0–56.5). The treatment was well tolerated by the patients, and no severe toxicities (CTCAE grade ≥ 3) were noted. Figure 1 shows a patient who underwent [^131^I]IMAZA therapy. Figure 2 summarizes the different theranostic targets in ACC and Figure 3 shows the corresponding radiopharmaceuticals.

## 3. Radiosynthesis of [^131^I]IMAZA

**Figure 4:**
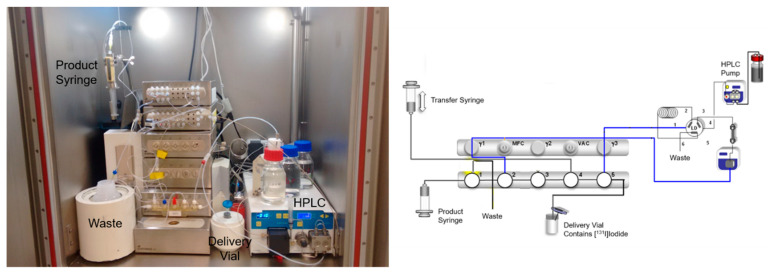
Photo and scheme of module for radiosynthesis of [^131^I]IMAZA.

The radiosynthesis and quality control of [^123/131^I]IMAZA for scintigraphy, dosimetry and therapy has already been published [55]. Here, destannylation reactions were used for labeling. Since this method yields the labeled products under very mild reaction conditions and with very high radiochemical yields, this method is frequently used and should be easily established in radiochemical laboratories that have experience with radioiodination. However, this does not apply to radioiodinations with > 30 GBq I-131, which are challenging in terms of radiation protection due to the high volatility of radioiodine in combination with the extremely high activity levels and the relatively high gamma energy of 364 keV. Therefore, labeling of [^131^I]IMAZA for endoradiotherapy had to be performed by an automated synthesis module (custom-made by Scintomics GmbH, Fürstenfeldbruck, Germany) inside a well-ventilated lead cell (see Figure 4).

To the delivery vial in which the [^131^I]iodide is dissolved in 1 mL 0.01 N NaOH (IBSSO; GE Healthcare, Braunschweig, Germany) were consecutively injected 5 mg trimethylstannylazetidinylamide in 1 mL ethanol, 120 µL 2 N hydrochloric acid and 2.25 mg chloramine T trihydrate in 150 µL water. The reaction solution was allowed to stand for three minutes. Thereafter, the reaction was quenched by adding 135 µL 2 N HCl and a solution of 4.50 mg Na_2_S_2_O_5_ in 150 µL water and the mixture was injected directly into the injection valve of the semi-preparative high-performance liquid chromatography system (HPLC) equipped with a RP-18 HPLC column (250 × 8 mm). An ethanol/phosphate buffer (40/60 *v*/*v*) mixture served as the HPLC solvent with a flow of 2.0 mL/min. Using typical starting activities of 34 GBq [^131^I]iodide, reproducibly > 25 GBq [^131^I]IMAZA were obtained, which were administered to the patients after successful quality control.

For each radiosynthesis, the exhaust air from the lead box was passed through activated carbon filters and checked for possible contamination. The personnel involved were monitored by means of personal dosimeters, finger ring dosimeters and a thyroid monitor. In all cases, only very low levels of contamination were detectable, so that the high-dose endoradiotherapies with [^131^I]IMAZA could be carried out safely. Regarding the radiosynthesis of the commercially available products [^177^Lu]Lu-DOTATATE, [^177^Lu]Lu-/[^90^Y]Y-PentixaTher and [^177^Lu]Lu-PSMA-617, please refer to the respective publications [58,59,60,61].

## 4. Future Perspectives

The investigations of patients with metastatic ACC with [^123^I]IMAZA showed an uptake in all known lesions (metastases and/or primary tumor) in only about 40% of the patients. This is likely due to dedifferentiation of the tumor cells resulting in low or no expression of the target enzymes CYP11B1 and CYP11B2. Therefore, only a minority of patients with high tracer uptake are candidates for subsequent endoradiotherapy with the analog [^131^I]IMAZA. Currently, alternative enzymatic and non-enzymatic targets with broader expression in ACC tissue are under investigation.

## 5. Summary

Adrenocortical carcinoma is a rare tumor entity and further therapeutic options in metastatic disease are desperately warranted. Several possible theranostic approaches exist, of which radiopharmaceuticals targeting specific enzymes of the adrenal cortex are currently the most promising and are the only theranostic radiopharmaceuticals ever used in patients to date. The theranostic twin [^123/131^I]IMAZA has shown good image quality and a good therapeutic effect in selected patients with advanced ACC, but cannot be used in all patients with ACC. Therefore, future developments are needed in order to provide a radiopharmaceutical with broader applications.

## Data Availability

Data sharing is not applicable.
